# Inflammation-Related DNA Damage and Cancer Stem Cell Markers in Nasopharyngeal Carcinoma

**DOI:** 10.1155/2016/9343460

**Published:** 2016-08-28

**Authors:** Shumin Wang, Ning Ma, Weilin Zhao, Kaoru Midorikawa, Shosuke Kawanishi, Yusuke Hiraku, Shinji Oikawa, Zhe Zhang, Guangwu Huang, Mariko Murata

**Affiliations:** ^1^Department of Environmental and Molecular Medicine, Mie University Graduate School of Medicine, Tsu, Mie, Japan; ^2^Department of Otolaryngology Head and Neck Surgery, First Affiliated Hospital of Guangxi Medical University, Nanning, China; ^3^Faculty of Nursing Science, Suzuka University of Medical Science, Suzuka, Mie, Japan; ^4^Faculty of Pharmaceutical Sciences, Suzuka University of Medical Science, Suzuka, Mie, Japan

## Abstract

Nitrative and oxidative DNA damage plays an important role in inflammation-related carcinogenesis. To investigate the involvement of stem cells in Epstein-Barr virus infection-related nasopharyngeal carcinoma (NPC), we used double immunofluorescence staining to examine several cancer stem/progenitor cell markers (CD44v6, CD24, and ALDH1A1) in NPC tissues and NPC cell lines. We also measured 8-nitroguanine formation as an indicator of inflammation-related DNA lesions. The staining intensity of 8-nitroguanine was significantly higher in cancer cells and inflammatory cells in the stroma of NPC tissues than in chronic nasopharyngitis tissues. Expression levels of CD44v6 and ALDH1A1 were significantly increased in cancer cells of primary NPC specimens in comparison to chronic nasopharyngitis tissues. Similarly, more intense staining of CD44v6 and ALDH1A1 was detected in an NPC cell line than in an immortalized nasopharyngeal epithelial cell line. In the case of CD24 staining, there was no significant difference between NPC and chronic nasopharyngitis tissues. 8-Nitroguanine was detected in both CD44v6- and ALDH1A1-positive stem cells in NPC tissues. In conclusion, CD44v6 and ALDH1A1 are candidate stem cell markers for NPC, and the increased formation of DNA lesions by inflammation may result in the mutation of stem cells, leading to tumor development in NPC.

## 1. Introduction

Chronic inflammation induced by infection has been postulated to be an important risk factor for various cancers [1, 2]. Epidemiological and experimental studies have provided evidence showing that chronic infection and inflammation contribute substantially to environmental carcinogenesis. During inflammation, reactive oxygen species (ROS) and reactive nitrogen species (RNS) are generated from inflammatory cells and are considered to play key roles in carcinogenesis [3]. Nitric oxide (NO) produced by inducible nitric oxide synthase (iNOS) reacts with superoxide anions (O_2_^•−^) from NAD(P)H oxidase to form various RNS, such as peroxynitrite (ONOO^−^), producing 8-oxo-7,8-dihydro-2′-deoxyguanosine (8-oxodG) and 8-nitroguanine [4]. 8-OxodG can be generated from other sources such as the mitochondrial respiratory chain. Therefore, 8-nitroguanine is a more specific biomarker for inflammation than 8-oxodG. Moreover, 8-nitroguanine is a potentially mutagenic DNA lesion and has been reported to play a significant role in and to be a biomarker for inflammation-related carcinogenesis [5].

Nasopharyngeal carcinoma (NPC) is a rare disease among Caucasians but one of the most prevalent malignant tumors and is the leading cause of death among all head and neck cancers in Southern China and Southeast Asia [6, 7]. Radiotherapy is the primary treatment, and concurrent chemoradiotherapy is the standard of care for advanced NPC [8, 9]. Since Epstein-Barr virus (EBV) infection is common and NPC is rare, it is a complex disease caused by the interaction of chronic EBV infection, environmental factors, and genetic and epigenetic changes, in a multistep process of carcinogenesis [7, 10–13]. Our previous study was the first to demonstrate 8-nitroguanine formation in the cancer cells of NPC patients via iNOS activation [14], showing that inflammation is an important risk factor for NPC development.

Recently, evidence has accumulated showing that stem cells are involved in inflammation-related carcinogenesis. According to the cancer stem cell hypothesis, not all tumor cells are involved in tumor evolution; rather, this property is limited to a subset of cells termed “cancer stem cells” [15, 16]. These cells are defined as tumor-initiating cells or rare cells with indefinite potential for self-renewal that drives tumorigenesis [15]. Moreover, several studies have shown that cancer cells have genetic instability, epigenetic changes, and an accumulation of mutations, suggesting that cancer is a genetic disease [16]. DNA lesions such as 8-nitroguanine and 8-oxodG with mutagenic properties occur in several types of inflammation-related cancer tissues [10]. Inflammation-associated tissue injury may activate stem/progenitor cells, and mutagenic stimuli from inflammation can accumulate multiple mutations and epigenetic changes in stem/progenitor cells [3, 10]. However, the developmental context of cancer stem cells is still not completely resolved issue.

Several reports suggest that CD24, CD44s including CD44v6, and ALDH1A1 are putative markers for cancer stem cells [17–22]. CD24 has been identified as a B-cell marker and found in NPC cells [23]. Yang et al. reported the identification of CD24 as a cancer stem cell marker in human NPC cell lines [24]. The combination of CD24 and CD44 as cancer stem cell markers showed controversial results in NPC. Several reports suggested an accumulation of CD24-negative and CD44-positive cells, as stemness characteristics in NPC [25, 26]. Another report showed that both CD24- and CD44-positive populations had stem-like properties under physiological Wnt/beta-catenin signaling [27]. Expression of CD44v6, a splicing variant of CD44, is associated with clinical significance by joint detection of CD62P in peripheral blood of NPC patients [28]. Several reports suggest that CD44v6 has more aggressiveness as the stem cell marker than CD44 [29, 30]. As a candidate molecular marker of cancer stem cells, ALDH1 has also drawn much attention in the field of NPC carcinogenesis [31–33]. ALDH1 is a zinc-containing cytosolic enzyme involved in the differentiation of various tissues and the induction of gene expression [17]. Recently, one member of the ALDH1 family, ALDH1A1, has been suggested as a marker of stem cells in several cancers [19, 20], including NPC [34]. Therefore, in this study, we focused on CD24, CD44v6, and ALDH1A1.

To investigate whether stem cells participate in inflammation-related carcinogenesis, we performed immunohistochemical (IHC) analysis to examine a nitrative DNA lesion (8-nitroguanine) and several stem cell markers (CD44v6, CD24, and ALDH1A1) in nasopharyngeal tissues obtained from patients with chronic nasopharyngitis or NPC. We also compared the expression of stem cell markers in an NPC cell line and an immortalized nasopharyngeal epithelial cell line by immunocytochemistry (ICC) analysis and flow cytometry.

## 2. Material and Methods

### 2.1. Patients

This study enrolled patients with NPC or chronic nasopharyngitis at the Department of Otolaryngology Head and Neck Surgery, First Affiliated Hospital of Guangxi Medical University, Nanning, China. Formalin-fixed and paraffin-embedded biopsy specimens were obtained from 28 patients (44.9 ± 10.0 years, 16 males, 12 females) with NPC, and chronic nasopharyngitis tissues were obtained from 14 patients (40.6 ± 11.6 years, 8 males, 4 females) with chronic nasopharyngitis, with the latter serving as normal controls. Patients provided informed consent prior to participation. All subjects' diagnoses were made by experienced pathologists according to the World Health Organization (WHO) classification. The pathological diagnosis of all NPC samples was nonkeratinizing carcinoma. This study was performed in accordance with ethical review committee approval notice (2009-07-07) of the First Affiliated Hospital of Guangxi Medical University, China, and ethical approval (number 1116) by Mie University, Japan. We removed identifying information from all samples before analysis.

Furthermore, a tissue array from US Biomax (Cat. number NPC961; Rockville, MD, USA) was analyzed to compare the levels of molecular markers. The tissue array provided 35 poorly differentiated nasopharyngeal squamous cell carcinoma tissues (42.5 ± 7.7 years, 30 males, 5 females) and 12 normal nasopharyngeal mucosal tissues (age and sex were not provided).

### 2.2. IHC Study

Double immunofluorescence was performed to examine the colocalization of 8-nitroguanine, CD44v6, and ALDH1A1. Rabbit polyclonal anti-8-nitroguanine antibody without cross reaction was produced as described previously [35]. Paraffin-embedded sections were incubated overnight at room temperature with the following primary antibodies: rabbit polyclonal anti-8-nitroguanine antibody (1 *μ*g/mL); mouse monoclonal anti-CD44v6 antibody (1 : 200, Abcam, Cambridge, MA, USA); goat polyclonal anti-ALDH1A1 antibody (1 : 200, Santa Cruz Biotechnology, Dallas, TX, USA). The sections were next incubated with the following fluorescent secondary antibodies at 1 : 400 each for 2 h at room temperature (Molecular Probes, Eugene, OR, USA: Alexa 488-labeled goat anti-mouse IgG antibody; Alexa 594-labeled goat anti-rabbit IgG antibody; Alexa 488-labeled donkey anti-mouse IgG antibody; Alexa 594-labeled donkey anti-goat IgG antibody). Finally, the nuclei were stained with 4′-6-diamidino-2-phenylindole (DAPI) and the sections were examined with a fluorescence microscope (BX53, Olympus, Tokyo, Japan). For immunoperoxidase study of CD24, standard immunoperoxidase methods were used to examine the distribution of CD24 in NPC tissues and normal controls. After deparaffinization and rehydration, antigen was retrieved in 5% urea buffer by microwave heating for 5 min and then incubation in 1% H_2_O_2_ for 30 min to block endogenous peroxidase activity. Sections of 3 *μ*m thickness were incubated overnight at room temperature with mouse monoclonal anti-CD24 (1 : 100, Abcam). The sections were incubated with biotinylated anti-mouse IgG for 3 h and then with avidin-biotin complex (Vectastain ABC kit, Vector Laboratories, Burlingame, CA, USA) for 2 h. Sections were then incubated with 3,3′-diaminobenzidine (DAB substrate kit; Vector Laboratories, Burlingame, CA, USA). Nuclei were counterstained by hematoxylin.

### 2.3. IHC Grading

IHC grading based on intensity and frequency of staining results was performed by 2 independent investigators without knowledge of the patients' clinicopathological features. The staining intensity was scored as negative (0), weak (+1), moderate (+2), or strong (+3). The frequency of positive cells in specific areas was scored as negative (0), less than 25% (+1), 25–50% (+2), 51–75% (+3), or more than 75% (+4). IHC grades were assigned by multiplying the intensity score by the frequency score, as follows: −, absent expression (0); +, weak expression (1–3); ++, moderate expression (4–6); +++, high expression (7–9); or ++++, very high expression (10–12).

### 2.4. Cell Culture

NPC cell line HK1 and immortalized nasopharyngeal epithelial cell line NP640 were the kind gifts of Professor Sai-Wah Tsao (Hong Kong University) [36, 37]. NPC cell line HK1 was maintained in RPMI 1640 medium (11875-093, Gibco) supplemented with 10% fetal bovine serum (S1820, Biowest, Nuaillé, France), 100 U/mL penicillin, and 100 *μ*g/mL streptomycin (15070-063, Gibco). Immortalization of nasopharyngeal epithelial cell NP460 was maintained in a 1 : 1 ratio of Defined Keratinocyte-SFM (DKSFM, Gibco) supplemented with growth factors and Epilife medium supplemented with growth factors EDGS (#S-012-5, Gibco), 100 U/mL penicillin, and 100 *μ*g/mL streptomycin. Cells were maintained at 37°C in a 5% CO_2_ incubator.

### 2.5. ICC Study

Cells were cultured overnight on culture slides (BD Falcon, Franklin Lakes, NJ, USA), with 2.5 × 10^4^ cells/500 *μ*L/well at 37°C in CO_2_ incubator. After culture for 48 h, the cells were fixed with 4% (v/v) formaldehyde in phosphate-buffered saline (PBS) for 10 min at room temperature and washed with PBS 3 times. The cells were treated with 1% (v/v) Triton X 100 for 20 min and then incubated with 5% (w/v) skim milk for 60 min at room temperature. Double immunofluorescence was performed to examine the colocalization of CD44v6 and ALDH1A1 on cells, by incubation with mouse monoclonal anti-CD44v6 (1 : 200, Abcam) and goat polyclonal anti-ALDH1A1 antibodies (1 : 200, Santa Cruz Biotechnology) overnight at room temperature. Then the cells were incubated for 2 h with the fluorescent secondary antibodies Alexa 488-labeled donkey anti-mouse IgG and Alexa 594-labeled donkey anti-goat IgG antibodies (1 : 400 each, Molecular Probes). The nuclei were stained with DAPI and the stained cells were examined under a florescent microscope (BX53, Olympus).

### 2.6. Western Blotting Analysis

Cells were treated with RIPA buffer on ice for 30 min and then sonicated for 15 s. The treated cells were centrifuged, and the protein concentration in the supernatant was measured with a Coomassie Protein Assay Reagent Kit (Pierce Biotechnology, Rockford, IL, USA). SDS-treated proteins were separated on SuperSep Ace, 5–20% polyacrylamide gels (Wako Pure Chemical Industries, Osaka, Japan), and transferred to PVDF membranes. Membranes were blocked with 5% skim milk in Tris-buffered saline (TBS) at room temperature for 5 h and incubated with primary antibody overnight at 4°C. Primary antibodies were used at the following concentrations (diluted with TBS): mouse anti-CD44v6 antibody (1 : 400, Abcam); goat anti-ALDH1A1 antibody (1 : 400, Santa Cruz Biotechnology); rabbit anti-GAPDH antibody (1 : 2500, Abcam). After incubation with a horseradish peroxidase-conjugated secondary antibody, the membranes were analyzed with the ECL Western Blotting Detection System (Amersham Biosciences) and then exposed to an X-ray film for detection. Specific bands were scanned by LAS 4000 mini (Fujifilm, Tokyo, Japan) and analyzed with ImageJ software, ver. 1.48.

### 2.7. Flow Cytometry Analysis of NPC Cells

For flow cytometry analysis the cells were harvested using 0.05% trypsin and 0.02% EDTA. After washing twice with PBS containing 0.5% BSA, cells were resuspended at a concentration of 10^6^ cells/mL in 4% formaldehyde solution for fixing and then permeabilized for intracellular staining using 0.1% Triton X 100 for 5 min. Cells were Fc-blocked by treatment with 1 *μ*g of human IgG (Invitrogen)/10^5^ cells for 15 min at room temperature prior to staining. APC-conjugated anti-human CD44v6 antibody (R&D Systems, Minneapolis, MN, USA) and FITC-conjugated anti-human ALDH1A1 antibody (Sino Biological, Beijing, China) were then added at a final concentration of 200 ng/mL and incubated for 25 min at 4°C in the dark with mixing. Fluorescence was measured with a flow cytometer (BD Biosciences, FACSCanto II, San Jose, CA, USA). Isotype-matched human antibodies (BD Biosciences) were used as controls.

### 2.8. Statistical Analysis

Statistical differences were determined by the chi-square test or Student's *t*-test. *P* < 0.05 was considered to be statistically significant. Statistical analysis was performed using SPSS19 for Windows.

## 3. Results

### 3.1. Nitrative DNA Damage in CD44v6-Positive Cancer Cells of NPC Biopsy Tissues

**Figure 1:**
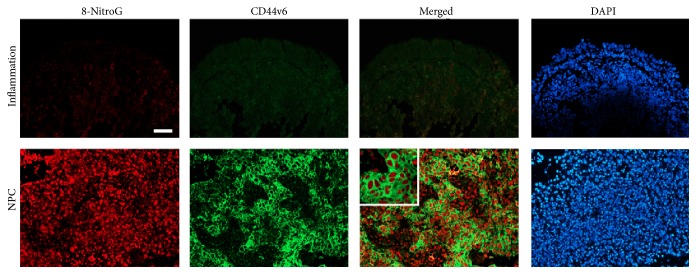
Double immunofluorescence staining of 8-nitroguanine and CD44v6 in chronic nasopharyngitis and NPC tissues. Formalin-fixed and paraffin-embedded biopsies of nasopharyngeal tissues were obtained from chronic nasopharyngitis (inflammation) and NPC tissues. The expression of 8-nitroguanine (red) and CD44v6 (green) were assessed by immunofluorescence staining. Nuclei were counterstained by DAPI (blue). Original magnification is 100x. The enlarged picture is shown in the inset. Scale bar represents 50 *μ*m.

We performed an immunofluorescence study to examine nitrative DNA lesions (8-nitroguanine) and the stem cell marker CD44v6 in nasopharyngeal tissues.  Figure 1 shows little or no immunoreactivity for 8-nitroguanine (8-NitroG, red) in chronic nasopharyngitis epithelium (Inflammation). Strong immunoreactivity was found in NPC tissues, primarily within the nuclei of cancer cells. Weak CD44v6 immunoreactivity (green) was observed in chronic nasopharyngitis epithelium. In contrast, CD44v6 showed intense staining in the cell membrane and also in the nuclear membranes of NPC cancer cells. 8-Nitroguanine was found in the same cells whose membranes were CD44v6-positive (merged image).

### 3.2. Nitrative DNA Damage in ALDH1A1-Positive Cancer Cells of NPC Biopsy Tissues

**Figure 2:**
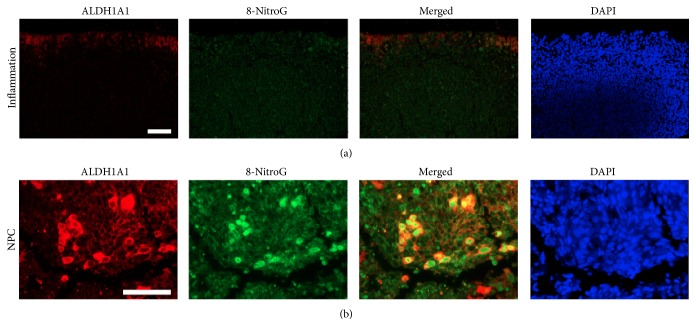
Double immunofluorescence staining of ALDH1A1 and 8-nitroguanine in chronic nasopharyngitis and NPC tissues. The expression of 8-nitroguanine (green) and ALDH1A1 (red) was assessed by immunofluorescence staining. Nuclei were counterstained by DAPI (blue). Original magnification is 100x (a) and 200x (b). Scale bar represents 50 *μ*m.

Figure 2 shows the expression patterns of the cancer stem cell marker ALDH1A1 (red) and 8-nitroguanine (8-NitroG, green) in primary NPC and chronic nasopharyngitis tissues. In chronic nasopharyngitis tissues (inflammation), epithelial cells showed weak immunofluorescence staining of ALDH1A1. In primary NPC tissues, ALDH1A1 was intensely expressed in the cytoplasm of NPC tumor cells. Strong immunoreactivity for 8-nitroguanine was observed in the cytoplasm as well as in the nuclei of cancer cells. The merged image indicated 8-nitroguanine formation in ALDH1A1-positive cells.

### 3.3. No Difference in Expression of CD24 in Nasopharyngeal Biopsy Tissues between Nasopharyngitis and NPC Patients

We also detected the expression patterns of another cancer stem cell marker, CD24, in primary NPC and inflammatory tissues (data not shown). CD24 showed intense staining of cell membranes in both inflammatory and tumor tissues.

### 3.4. Formation of 8-Nitroguanine and Expression of Cancer Stem Cell Markers in NPC

We analyzed the IHC grade data derived from biopsy samples from patients in southern China. Additionally, samples in the NPC tissue array (US Biomax, Cat. number NPC961; Rockville, MD, USA) were analyzed with the same methods. The results are summarized in Table 1. The IHC grade of 8-nitroguanine was significantly higher in NPC than in biopsy nasopharyngitis tissues. Also, a significant difference was observed among the tissue array samples. There were no significant differences between the nasopharyngitis and normal mucosa samples or between the two NPC groups (biopsy and array). Cancer stem cell markers (CD44v6 and ALDH1A1) were more highly expressed in the biopsy NPC tissues compared with the nasopharyngitis. Similar results were obtained between array NPC tissues and normal mucosa tissues. CD24 showed no significant difference between NPC and nasopharyngitis in biopsy samples and also between NPC and normal tissues in array samples. Interestingly, CD44v6 and CD24 had significantly higher expression in NPC of biopsy than NPC of array, but ALDH1A1 did not.

### 3.5. CD44v6 and ALDH1A1 Double-Positive Cancer Cells in NPC Biopsy Tissues

**Figure 3:**
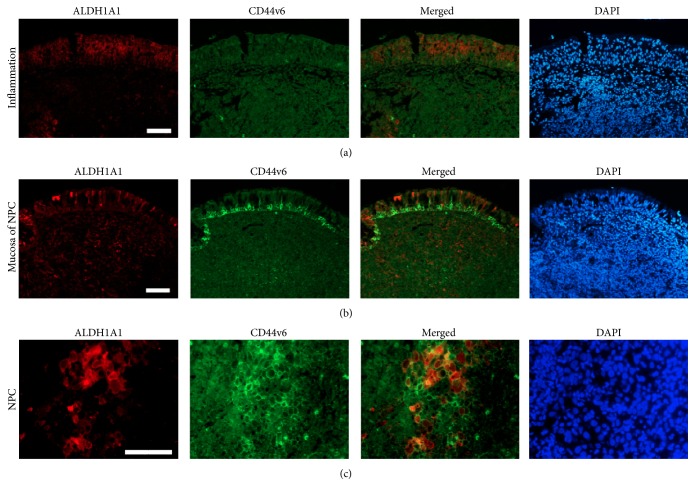
Double immunofluorescence staining of ALDH1A1 and CD44v6 in chronic nasopharyngitis and NPC tissues. The expression of CD44v6 (green) and ALDH1A1 (red) was assessed by immunofluorescence staining. Nuclei were counterstained by DAPI (blue). Original magnification is 100x (a, b) and 200x (c). Scale bar represents 50 *μ*m.

Figure 3 shows the double immunofluorescence staining of ALDH1A1 (red) and CD44v6 (green) in chronic nasopharyngitis (inflammation) and NPC tissues. Weak immunofluorescence staining of ALDH1A1 and CD44v6 was observed in chronic nasopharyngitis epithelium (a and b). In contrast, strong immunoreactivities of ALDH1A1 and CD44V6 were observed in the tumor cells (c). ALDH1A1 and CD44v6 double-positive cells were observed in the cancer nest in the merged image.

### 3.6. CD44v6 and ALDH1A1 Double-Positive Cancer Cells in Nasopharyngeal Cell Lines

**Figure 4:**
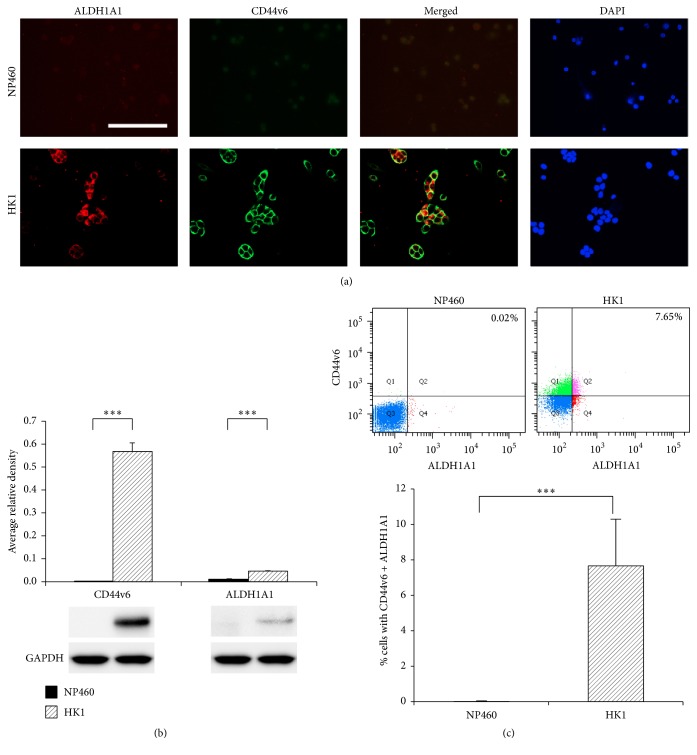
Expression of CD44v6 and ALDH1A1 in NP460 cells and HK1 cells. (a) Double immunofluorescence staining of CD44v6 (green) and ALDH1A1 (red) in a normal nasopharyngeal cell line NP460 and an NPC cell line HK1. Nuclei were counterstained by DAPI (blue). Original magnification is 400x. Scale bar represents 50 *μ*m. (b) CD44v6 or ALDH1A1 levels in HK1 cells in comparison with NP460 cells by Western blot analysis (*n* = 4). Expression levels of target proteins (CD44v6 and ALDH1A1) were normalized against the corresponding levels of GAPDH. (c) HK1 and NP460 were stained with CD44v6 and ALDH1A1 and subjected to flow cytometry analysis (*n* = 6). Data are shown as mean ± standard deviation. *P* values were calculated using Student's *t*-test (^*∗∗∗*^ *P* < 0.001).

Figure 4(a) shows the double immunofluorescence staining of ALDH1A1 (red) and CD44v6 (green) in normal nasopharyngeal cell line NP460 cells and NPC cell line HK1 cells. Little or no immunofluorescent staining of ALDH1A1 and CD44v6 was observed in the NP460 cells. In contrast, ALDH1A1 was expressed in the cytoplasm of HK1 cells. CD44v6 was stained in the cell membrane and also in the nuclear membrane of HK1 cells. A few ALDH1A1 and CD44V6 double-positive cells were observed in the merged image. Western blotting confirmed the differences in expression levels of CD44v6 and ALDH1A1 between NP460 cells and HK1 cells (Figure 4(b), *P* < 0.001). Stem cell markers, CD44v6 and ALDH1A1, were analyzed by flow cytometry in NP460 cells and HK1 cells. The percentage of CD44v6 and ALDH1A1 double-positive cells in HK1 cells was 7.65%, but these cells were hardly detected in NP460 cells (Figure 4(c), *P* < 0.001).

## 4. Discussion

Accumulating evidence in recent years strongly indicates that stem/progenitor cells are involved in inflammation-mediated carcinogenesis [38, 39]. The present IHC analyses semiquantitatively confirmed that the expression levels of both CD44v6 and ALDH1A1 were increased in NPC tissues in comparison with chronic nasopharyngitis tissues. Western blot analysis confirmed that CD44v6 and ALDH1A1 were highly expressed in an NPC cell line compared with a normal nasopharyngeal epithelial cell line. Double immunostaining of CD44v6 and ALDH1A1 was occasionally observed in cancer nest cells and the NPC cell line. Todaro et al. reported that CD44v6 was a marker of constitutive and reprogrammed cancer stem cells driving cancer metastasis [22]. Hou et al. indicated that increased expression of ALDH1A1 in NPC was associated with enhanced invasiveness [34]. These reports and our data led us to hypothesize that NPC may be a disease which is related with stem/progenitor cells.

It is assumed that chronic inflammation, such as that observed in chronic nasopharyngitis, can be an etiological factor of human cancers. Inflammation-induced tissue injury activates stem/progenitor cells, and these cells are damaged under ROS/RNS-rich environment. Mutated stem/progenitor cells would proliferate leading to cancer development. Our previous reports also confirmed that DNA damage, including 8-nitroguanine formation, could be used as a biomarker to evaluate the risk of EBV-mediated NPC [14, 40]. The present study revealed that the stem/progenitor markers CD44v6 and ALDH1A1 were positively stained in the NPC cases. We demonstrated that the amount of 8-nitroguanine in CD44v6- or ALDH1A1-positive tissues was significantly higher in NPC than in inflammatory tissues. 8-Nitroguanine, like 8-oxodG, is known to cause G:C to T:A transversions [41]. In our previous studies we proposed a mechanism for the generation of cancer stem cells by inflammation [3, 42, 43]. Interestingly, the nuclear localization of COX-2 was significantly associated with the upregulation of CD44v6 in sporadic bladder cancer tissues [42], suggesting that the stemness marker has some relation with inflammation. Our present results indicate that CD44v6- and/or ALDH1A1-positive cells are damaged under nitrative stress, and accumulation of mutagenic DNA lesions may play a role in NPC carcinogenesis.

The present study revealed that moderate immunofluorescence staining of ALDH1A1 was observed in chronic nasopharyngitis epithelium. In contrast, ALDH1A1 was stained intensely in the cytoplasm of NPC cancer cells, the same location where 8-nitroguanine was formed in ALDH1A1-positive cells. CD44v6 was observed in NPC tissues and the NPC cell line but showed weak staining in inflammatory tissues and the immortalized nasopharyngeal epithelial cell line. In the present study, double immunofluorescence staining demonstrated that a small number of cells coexpressed these stem/progenitor cell markers. In the RNS-rich microenvironment, ALDH1A1-positive stem cells with nitrative DNA damage might become cancer stem cells expressing CD44v6, which plays a major role in stem cell maintenance and nuclear reprogramming. Flow cytometry analysis of CD44v6 and ALDH1A1 stem cell markers in NP460 cells and HK1 cells in our study indicated that ALDH1A1- and CD44v6-positive cells accounted for only 7.65% of the total number of cells in the NPC cell line. The cancer stem cell population consists of a very small fraction of the total population that simultaneously expresses a set of defined markers [44]. The present results showed that a small number of cells had both CD44v6 and ALDH1A1 immunoreactivity. Expression of CD44v6 and CD24 in NPC biopsy samples in endemic area was higher than NPC array samples in nonendemic area. Interestingly, in the Chinese that have migrated to North America, the incidence of NPC was reduced but still higher than in the background population [6]. Environmental factors may have some effects on virus activation resulting in the difference of CD44v6 and CD24 expression between two groups. Relevantly, several studies demonstrated that CD44v6 expression was increased as human papillomavirus-associated cervical carcinoma progressed to more advanced clinical stages [45], and serum levels of CD44v6 were increased in patients with human immunodeficiency virus-related non-Hodgkin's lymphoma [46]. CD24 polymorphism is associated with viral clearance and risk for chronic hepatitis virus infection [47, 48]. In the present study, double immunofluorescence staining of these stem/progenitor cell markers showed that a small number of cells coexpressed CD44v6 and ALDH1A1, which may be indicators of cancer stem cells in NPC.

Our data indicated that 8-nitroguanine was formed in CD44v6- and/or ALDH1A1-positive stem cells in NPC tissues, suggesting that inflammation may increase the number of mutant stem cells that participate in NPC development. The present study shows that cancer cells are the progeny of cancer stem cells and become polyclonal due to irregular progenitor cell differentiation caused by alteration of genes involved in cell differentiation. Therefore, NPC may be a disease of stem/progenitor cells that can be detected by the formation of 8-nitroguanine in combination with increased expression of stem/progenitor markers. Understanding of molecular markers of cancer stem cells in NPC can improve the development of clinical strategies, as seen in inhibition of tumor growth by interference with CD44v6 signaling [49] and a specific ALDH1A1 inhibitor for cancer stem cell target therapy [50]. In addition, higher serum CD44v6 levels in lung cancer [51] and joint detection of CD44v6 and CD62P in NPC peripheral blood were found to be significantly unfavorable prognostic factors. Therefore, further studies are needed to apply cancer stem cell markers for therapeutic targets and biomarkers in NPC.

## Figures and Tables

**Table 1:** IHC grades of biomarkers in nasopharynx samples.

8-Nitroguanine IHC grades		−	+	++	+++	++++	*P* value	
Biopsy	Nasopharyngitis (12)	4	6	2	0	0		
	NPC (26)	0	5	8	10	3	0.001	

Array	Normal mucosa (12)	6	5	1	0	0		0.662
	NPC (35)	1	3	10	8	13	0.000	0.135

CD44v6 IHC grades		−	+	++	+++	++++	*P* value	

Biopsy	Nasopharyngitis (11)	7	4	0	0	0		
	NPC (18)	0	2	3	5	8	0.002	

Array	Normal mucosa (12)	11	1	0	0	0		0.262
	NPC (35)	6	4	15	9	1	0.001	0.001

ALDH1A1 IHC grades		−	+	++	+++	++++	*P* value	

Biopsy	Nasopharyngitis (5)	2	2	1	0	0		
	NPC (16)	0	3	5	6	2	0.044	

Array	Normal mucosa (12)	4	6	2	0	0		0.932
	NPC (35)	0	3	11	11	10	0.000	0.514

CD24 IHC grades		−	+	++	+++	++++	*P* value	

Biopsy	Nasopharyngitis (9)	0	3	4	2	0		
	NPC (10)	0	3	2	5	0	0.386	

Array	Normal mucosa (12)	2	7	3	0	0		0.143
	NPC (35)	15	13	4	3	0	0.189	0.006

IHC grades were assigned to each specimen according to the grade of staining intensity as described in the Materials and Methods.

*P* values were calculated by the chi-square test when comparing NPC and non-cancer tissues (nasopharyngitis, normal mucosa) in each group (biopsy or array, left). Comparisons were also made between biopsy nasopharyngitis tissues and array normal mucosa (right upper) and between biopsy NPC and array NPC (right lower).

## Abbreviations

NPC: Nasopharyngeal carcinoma
IHC: Immunohistochemistry
ICC: Immunocytochemistry
CD44v6: Splicing variant of CD44
CD44: Cluster of differentiation 44
ALDH1A1: Aldehyde dehydrogenase 1 family, member A1
CD24: Clusters of differentiation 24
ROS: Reactive oxygen species
RNS: Reactive nitrogen species
iNOS: Inducible nitric oxide synthase.
